# Crystal structure and Hirshfeld surface analysis of 3-cyano-4-hy­droxy-2-(4-methyl­phen­yl)-6-oxo-*N*-phenyl-4-(thio­phen-2-yl)cyclo­hexane-1-carbox­amide 0.04-hydrate

**DOI:** 10.1107/S2056989021002449

**Published:** 2021-03-09

**Authors:** Farid N. Naghiyev, Victor N. Khrustalev, Mehmet Akkurt, Elnur Z. Huseynov, Ali N. Khalilov, Anzurat A. Akobirshoeva, İbrahim G. Mamedov

**Affiliations:** a Department of Chemistry, Baku State University, Z. Khalilov str. 23, Az, 1148 Baku, Azerbaijan; b Peoples’ Friendship University of Russia (RUDN University), Miklukho-Maklay St.6, Moscow, 117198 , Russian Federation; c N. D. Zelinsky Institute of Organic Chemistry RAS, Leninsky Prosp. 47, Moscow, 119991 , Russian Federation; d Department of Physics, Faculty of Sciences, Erciyes University, 38039 Kayseri, Turkey; e "Composite Materials" Scientific Research Center, Azerbaijan State Economic University (UNEC), H. Aliyev str. 135, Az 1063, Baku, Azerbaijan; f Acad Sci Republ Tadzhikistan, Kh Yu Yusufbekov Pamir Biol Inst, 1 Kholdorova St, Khorog 736002, Gbao, Tajikistan

**Keywords:** crystal structure, cyclo­condensation product, 1,2,7,8-tetra­hydro­iso­quinoline ring system, Hirshfeld surface analysis

## Abstract

In the title compound, C_25_H_22_N_2_O_3_S·0.04H_2_O, the central cyclo­hexane ring adopts a chair conformation. In the crystal, mol­ecules are linked by N—H⋯O, C—H⋯O, and C—H⋯N hydrogen bonds, forming the mol­ecular layers parallel to the *bc* plane, which inter­act by the van der Waals forces between them. A Hirshfeld surface analysis indicates that the contributions from the most prevalent inter­actions are H⋯H (41.2%), C⋯H/H⋯C (20.3%), O⋯H/H⋯O (17.8%) and N⋯H/H⋯N (10.6%).

## Abstract 2

In the crystal structure, mol­ecules are linked by N—H⋯O, C—H⋯O and C—H⋯N hydrogen bonds, forming mol­ecular layers parallel to the *bc* plane, which inter­act by the van der Waals forces between them.

## Chemical context   

The significance of β-carbonyl compounds in organic chemistry is difficult to overestimate. They are valuable building blocks in organic synthesis and coordination complexes (Shokova *et al.*, 2015 ▸; Ma *et al.*, 2015 ▸; Gurbanov *et al.*, 2017 ▸, 2018 ▸; Mittersteiner *et al.*, 2020 ▸). Cyclo­condensation reactions of *β*-diketones with various reagents mainly lead to the formation of carbocyclic and heterocyclic compounds (Mamedov *et al.*, 2013 ▸, 2019 ▸; Naghiyev *et al.*, 2019 ▸; Naghiyev, 2020 ▸). Being a carbocyclic system, cyclo­hexa­none derivatives are scaffolds in many synthetic and natural products. They possess a broad spectrum of biological assets, such as anthelmintic, anti-inflammatory, anti­bacterial, anti­cancer, anti­convulsant, anti­tubercular, anti­tumor, anti­leukemic, anti­viral, analgesic, herbicidal and enzyme inhibitory activities (Holland *et al.*, 1990 ▸; Fu & Ye, 2004 ▸; Liu *et al.*, 2009 ▸; Gein *et al.*, 2015 ▸; Mamedov *et al.*, 2017 ▸; Nosova *et al.*, 2020 ▸). The methods used most widely for the synthesis of these functionalized cyclo­hexa­nones involve the condensation of aldehydes with β-carbonyl compounds (Gein *et al.*, 2015 ▸; Nosova *et al.*, 2020 ▸).

As part of our studies on the chemistry of β-dicarbonyl compounds, as well as taking into account our ongoing structural studies (Naghiyev, Akkurt *et al.*, 2020 ▸; Naghiyev, Cisterna *et al.*, 2020 ▸; Naghiyev, Mammadova *et al.*, 2020 ▸; Naghiyev *et al.*, 2021 ▸), we report here the crystal structure and Hirshfeld surface analysis of the title compound, 3-cyano-4-hy­droxy-2-(4-methyl­phen­yl)-6-oxo-*N*-phenyl-4-(thio­phen-2-yl)-cyclo­hexane-1-carboxamide 0.04-hydrate.
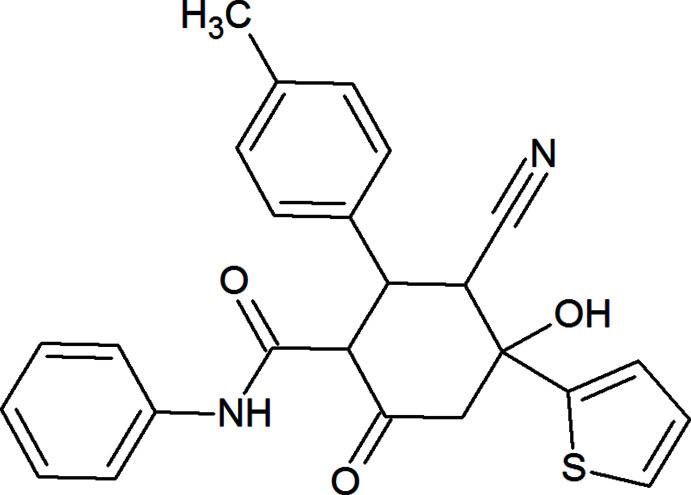


## Structural commentary   

**Figure 1:**
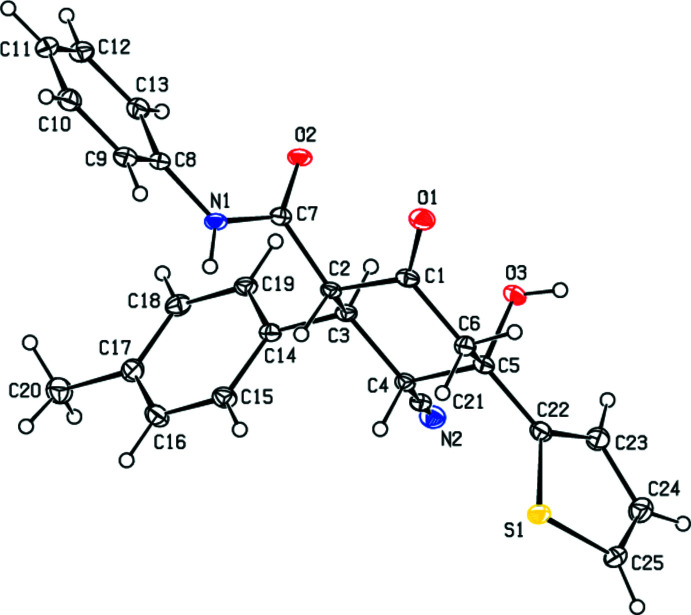
The mol­ecular structure of the title compound. Displacement ellipsoids are drawn at the 30% probability level.

In the title compound, (Fig. 1 ▸), the central cyclo­hexane ring (C1–C6) adopts a chair conformation with puckering parameters (Cremer & Pople, 1975 ▸) *Q*_T_ = 0.570 (2) Å, θ = 5.1 (2)° and φ = 226 (2)°. The thio­phene (S1/C22–C25), phenyl (C8–C13) and benzene (C14–C19) rings make dihedral angles of 68.05 (10), 46.41 (9) and 87.95 (10)°, respectively, with the mean plane of the central cyclo­hexane ring. The thio­phene ring forms dihedral angles of 21.88 (10) and 73.64 (10)°, respectively, with the phenyl and benzene rings, which subtend a dihedral angle of 80.91 (10)°. The C2—C7—N1—C8 torsion angle is 178.99 (18)°.

## Supra­molecular features   

**Figure 2:**
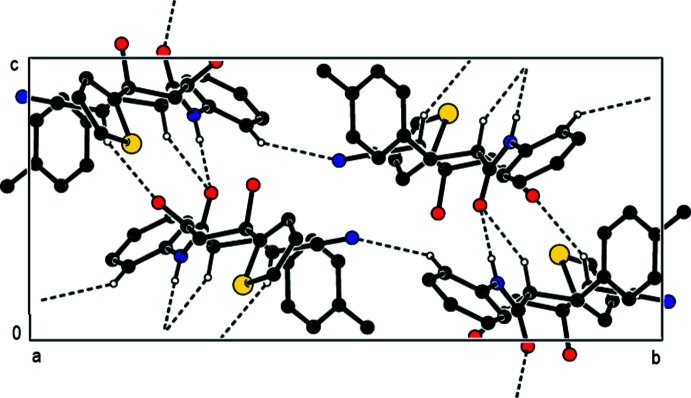
A view down the *a* axis of the inter­molecular N—H⋯O, C—H⋯O and C—H⋯N hydrogen bonds of the title compound.

**Figure 4:**
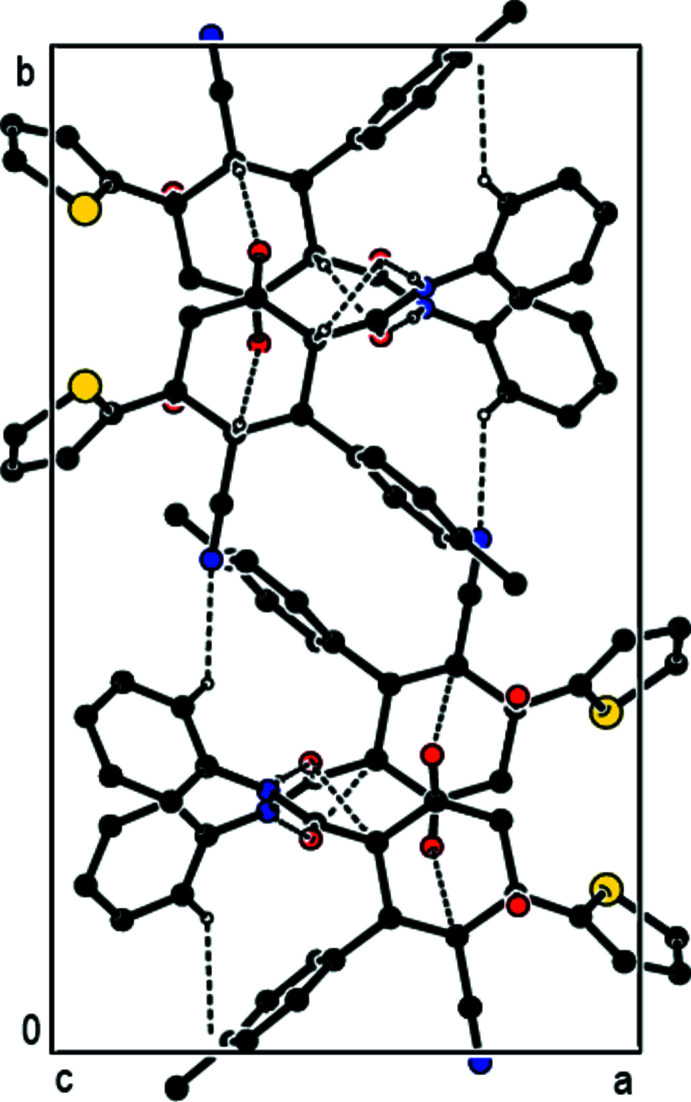
A view down the *c* axis of the inter­molecular N—H⋯O, C—H⋯O and C—H⋯N hydrogen bonds of the title compound.

In the crystal, N—H⋯O and C—H⋯O hydrogen bonds link adjacent mol­ecules, forming mol­ecular ribbons with 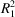
(6) and 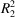
(10) ring motifs (Bernstein *et al.*, 1995 ▸) along the *c-*axis direction (Table 1 ▸; Figs. 2 ▸ and 3 ▸). These ribbons are linked by weak C—H⋯N non-classical hydrogen bonds, forming layers of mol­ecules parallel to the *bc* plane (Table 1 ▸; Fig. 4 ▸), with only van der Waals inter­actions between them.

## Hirshfeld surface analysis   

**Figure 5:**
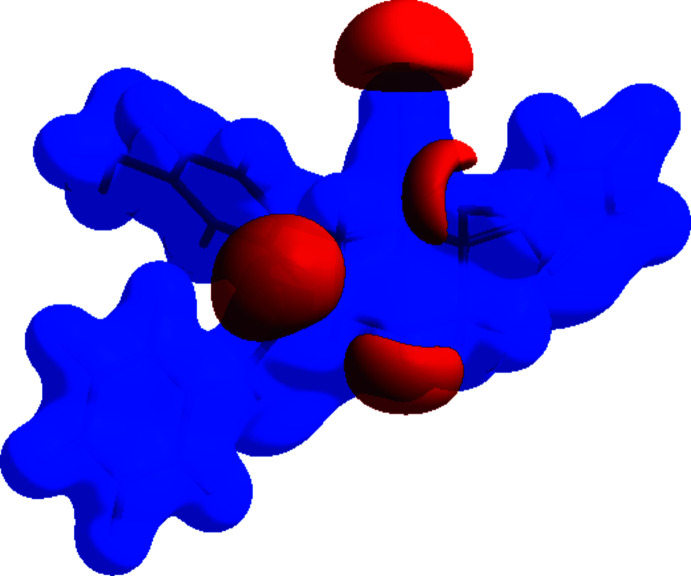
The Hirshfeld surface of the title compound plotted over electrostatic potential energy in the range from −0.0500 to 0.0500 a.u. using the *STO*-3 *G* basis set at the Hartree–Fock level of theory. Hydrogen-bond donors and acceptors are shown as blue and red regions around the atoms, corresponding to positive and negative potentials, respectively.

The Hirshfeld surface for the title compound and its associated two-dimensional fingerprint plots were calculated using *CrystalExplorer17* (Turner *et al.*, 2017 ▸). The oxygen atom of the water mol­ecule with a low occupancy factor of about 4% was not taken into account in the process. The Hirshfeld surface mapped over electrostatic potential (Spackman *et al.*, 2008 ▸; Jayatilaka *et al.*, 2005 ▸) is shown in Fig. 5 ▸. The blue regions indicate positive electrostatic potential (hydrogen-bond donors), while the red regions indicate negative electrostatic potential (hydrogen-bond acceptors).

**Figure 6:**
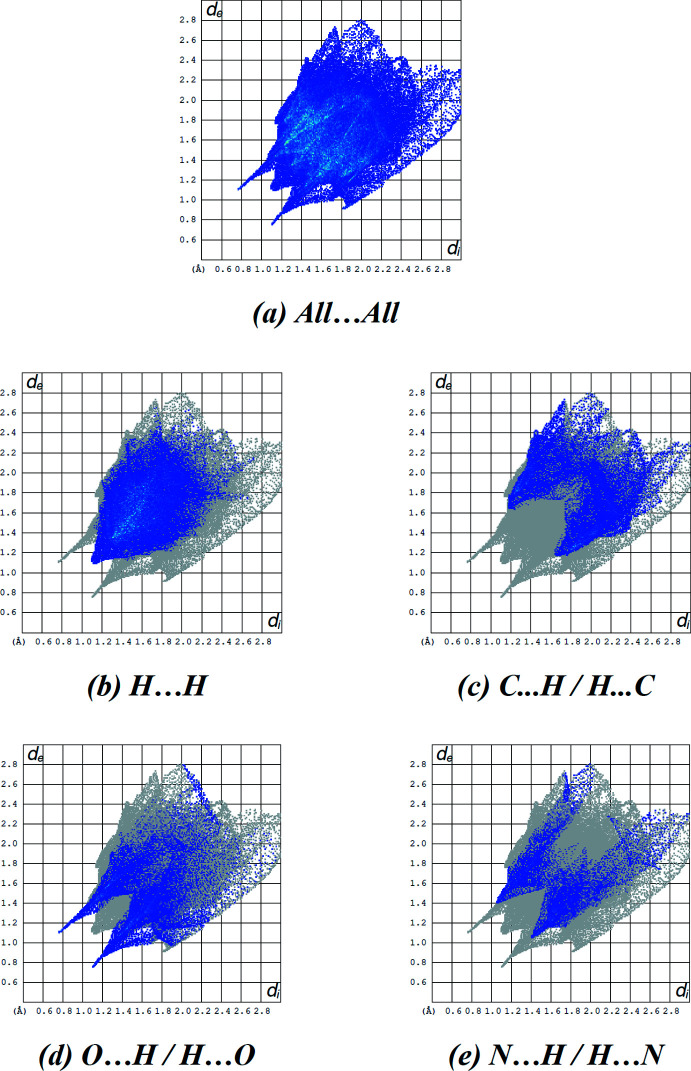
The two-dimensional fingerprint plots of the title compound, showing (*a*) all inter­actions, and delineated into (*b*) H⋯H, (*c*) C⋯H/H⋯C, (*d*) O⋯H/H⋯O and (*e*) N⋯H/H⋯N, inter­actions [*d*_e_ and *d*_i_ represent the distances from a point on the Hirshfeld surface to the nearest atoms outside (external) and inside (inter­nal) the surface, respectively].

The overall two-dimensional fingerprint plot, and those delineated into H⋯H (41.2%), C⋯H/H⋯C (20.3%), O⋯H/H⋯O (17.8%) and N⋯H/H⋯N (10.6%) contacts are illustrated in Fig. 6 ▸ *a*–*e*, respectively. The other minor contributions to the Hirshfeld surface are from S⋯H/H⋯S (5.5%), O⋯O (1.9%), C⋯C (1.1%), S⋯C/C⋯S (1.0%), O⋯C/C⋯O (0.5%) and O⋯N/N⋯O (0.1%) contacts. The large number of H⋯H, C⋯H/H⋯C, O⋯H/H⋯O and N⋯H/ H⋯N inter­actions suggest that van der Waals inter­actions and hydrogen bonding play the major roles in the crystal packing (Hathwar *et al.*, 2015 ▸).

## Database survey   

A search of the Cambridge Structural database (CSD, version 5.42, update November 2020; Groom *et al.*, 2016 ▸) for the 4-hy­droxy-5-methyl-2-oxo­cyclo­hexane-1-carboxamide moiety revealed seven hits, of which the structures most similar to the that of the title compound are 4-hy­droxy-4,*N*,*N*′-trimethyl-2-(3-nitro­phen­yl)-6-oxo-1,3-cyclo­hexa­nedicarbox-amide (HALROB; Ravikumar & Mehdi, 1993 ▸), 4-hy­droxy-*N*,*N*,*N*′,*N*′,4-penta­methyl-6-oxo-2-phenyl­cyclo­hexane-1,3-di­carboxamide (IFUDOD; Gein *et al.*, 2007 ▸), 5-hy­droxy-5-methyl-3-phenyl-2,4-bis­(*N*-methyl­carbamo­yl)cyclo­hexa­none (IWEVOV; Mohan *et al.*, 2003 ▸), 5-hy­droxy-5-methyl-3-(*o*-tol­yl)-2,4-bis­(*N*-methyl­carbamo­yl)cyclo­hexa­none (IWEVUB; Mohan *et al.*, 2003 ▸), 2-(4-chloro­phen­yl)-4-hy­droxy-4-methyl-6-oxo-*N*,*N*′-di­phenyl­cyclo­hexane-1,3-dicarboxamide *N*,*N*-di­methyl­formamide solvate (OZUKAX; Tkachenko *et al.*, 2014 ▸), 4-hy­droxy-4-methyl-2-(4-methyl­phen­yl)-6-oxo-*N* ^1^,*N* ^3^-di­phenyl­cyclo­hexane-1,3-dicarboxamide (PEWJUZ; Fatahpour *et al.*, 2018 ▸) and 4-hy­droxy-4-methyl-2-(3-nitrophen­yl)-6-oxo­cyclo­hexane-1,3-dicarboxamide ethanol solvate (ZOMDUD; Gein *et al.*, 2019 ▸).

ZOMDUD crystallizes in the monoclinic space group *P*2_1_/*c*, with *Z* = 4, HALROB, IFUDOD and IWEVUB in *P*2_1_/*n* with *Z* = 4, PEWJUZ in *I*2/*c* with *Z* = 4, and IWEVOV and OZUKAX in the ortho­rhom­bic space group *Pbca* with *Z* = 8.

In the crystal of HALROB, the amide carbonyl groups are oriented in different directions with respect to the cyclo­hexa­none ring. These orientations of the carboxamide groups facilitate the formation of an intra­molecular O—H⋯O hydrogen bond. The mol­ecules are packed such that chains are formed along the *b*-axis direction. These chains are held together by N—H⋯O hydrogen bonds.

In the crystal IFUDOD, there are no classical hydrogen bonds. Inter­molecular C—H⋯O contacts and weak C—H⋯π inter­actions lead to the formation of a three-dimensional network.

In the crystal of IWEVOV, the mol­ecules pack such that both carbonyl O atoms, participate in hydrogen-bond formation with symmetry-related amide nitro­gen atoms, present in the carbamoyl substituents, forming N—H⋯O hydrogen bonds in a helical arrangement. In the crystal, the phenyl rings are positioned so as to favour edge-to-edge aromatic stacking. When the crystal packing is viewed normal to the *ac* plane, it reveals a ‘wire-mesh’ type hydrogen-bond network.

In the crystal of IWEVUB, unlike in IWEVOV where both carbonyl O atoms participate in hydrogen bonding, only one of the carbonyl oxygen atoms participates in inter­molecular N—H⋯O hydrogen bonding while the other carbonyl oxygen participates in a weak C—H⋯O inter­action. In addition, one of the amide nitro­gen atoms participates in N—H⋯O hydrogen bonding with the hydroxyl oxygen atom, linking the mol­ecules in a helical arrangement, which is similar to that in the structure of IWEVOV. As observed in the structure of IWEVOV, the packing of the mol­ecules viewed normal to the *ab* plane resembles a ‘wiremesh’ arrangement of the mol­ecules.

In OZUKAX, mol­ecules are linked by inter­molecular N—H⋯O and C—H⋯O hydrogen bonds, forming sheets parallel to the *ac* plane. C—H⋯π inter­actions are also observed. Inter­molecular O—H⋯O hydrogen bonds consolidate the mol­ecular conformation.

In PEWJUZ, mol­ecules are linked by inter­molecular N—H⋯O and C—H⋯O hydrogen bonds, forming sheets parallel to the *bc* plane. C—H⋯π inter­actions are also observed.

In ZOMDUD, mol­ecules are linked by inter­molecular N—H⋯O and C—H⋯O hydrogen bonds, forming a three-dimensional network. C—H⋯π inter­actions are also observed.

Inter­molecular inter­actions can be weaker or more robust based on the presence or absence of different functional groups and the mol­ecular environment, depending on the crystal system, which all affect the mol­ecular conformation.

## Synthesis and crystallization   

To a dissolved mixture of 2-(thio­phene-2-carbon­yl)-3-(*p*-tol­yl)acrylo­nitrile (1.32 g; 5.2 mmol) and acetoacetanilide (0.92 g; 5.2 mmol) in methanol (35 mL), 2–3 drops of methyl piperazine were added and the mixture was stirred at room temperature for 5–7 min. The reaction mixture was kept in a closed flask for 24–48 h. Then, 25 mL of methanol was removed from the reaction mixture and it was left overnight. The precipitated needle-like crystals were separated by filtration and recrystallized from ethanol (yield 72%; m.p. 483–484 K).

^1^H NMR (300 MHz, DMSO-*d*_6_, m.h.): δ 2.23 (*s*, 3H, C*H*_3_); 2.79 (*d*, 2H, C*H*_2_, ^2^ *J*_H-H_ = 18.1 Hz); 3.50 (*t*, 1H, C*H*, ^3^ *J*_H-H_ = 13.8 Hz); 3.63 (*s*, 1H, O*H*); 4.06 (*d*, 1H, C*H*, ^3^ *J*_H-H_ = 10.5 Hz); 4.28 (*dd*, 1H, C*H*, ^3^ *J*_H-H_ = 10.5 Hz, ^3^ *J*_H-H_ = 11.9 Hz); 6.97–7.48 (*m*, 12H, 9Ar-*H* + 3C*H*_thien­yl_); 9.94 ppm (*s*, 1H, N*H*). ^13^C NMR (75 MHz, DMSO-*d*_6_, m.h.): δ 21.14 (*C*H_3–_-Ar), 44.26 (*C*H—Ar), 47.40 (*C*H—CN), 54.07 (*C*H_2_), 62.64 (C*H*—CO), 75.29 (O—*C*_quat._), 119.02 (*C*N), 119.49 (2*C*H_arom_), 123.87 (*C*H_thien­yl_), 124.45 (*C*H_arom_), 125.71 (*C*H_thien­yl_), 127.63 (*C*H_thien­yl_), 128.75 (2 *C*H_arom_), 129.14 (2 *C*H_arom_),129.54 (2 *C*H_arom_), 137.06 (*C*_arom_), 137.17 (*C*_arom_), 139.14 (*C*_arom_), 150.57 (*C*_thien­yl_), 165.85 (O=*C*), 203.12 ppm (O=*C*_ket_). As a result of the overlap of peaks in the ^1^H NMR spectrum, it was not possible to determine precisely all coupling constants.

## Refinement   

Crystal data, data collection and structure refinement details are summarized in Table 2 ▸. The H atoms of the OH and NH groups were located from the difference-Fourier synthesis and refined freely. All C-bound H atoms were positioned geometrically and refined using a riding model, with C—H = 0.95–1.00 Å, and with *U*_iso_(H) = 1.2 or 1.5*U*_eq_(C).

Data with a resolution higher than 0.8 Å have a mean *I*/σ(*I*) of less than 4, and significant errors in the equivalent intensities (high *R*_merge_). The dataset was therefore truncated at 0.8 Å. Furthermore, there is a small cavity in the crystal, which is only partially occupied by a water mol­ecule (only about 4%) and the protons could not be located. It is also highly probable that, in the presence of a fully occupied water mol­ecule, the proton of the OH group would have a different orientation.

## Supplementary Material

Crystal structure: contains datablock(s) I. DOI: 10.1107/S2056989021002449/jq2005sup1.cif

Structure factors: contains datablock(s) I. DOI: 10.1107/S2056989021002449/jq2005Isup2.hkl

13C NMR spectrum. DOI: 10.1107/S2056989021002449/jq2005sup3.jpg

1H NMR spectrum. DOI: 10.1107/S2056989021002449/jq2005sup4.jpg

Supporting information file. DOI: 10.1107/S2056989021002449/jq2005Isup5.cml

CCDC reference: 2068003

Additional supporting information:  crystallographic information; 3D view; checkCIF report

## Figures and Tables

**Figure 3:**
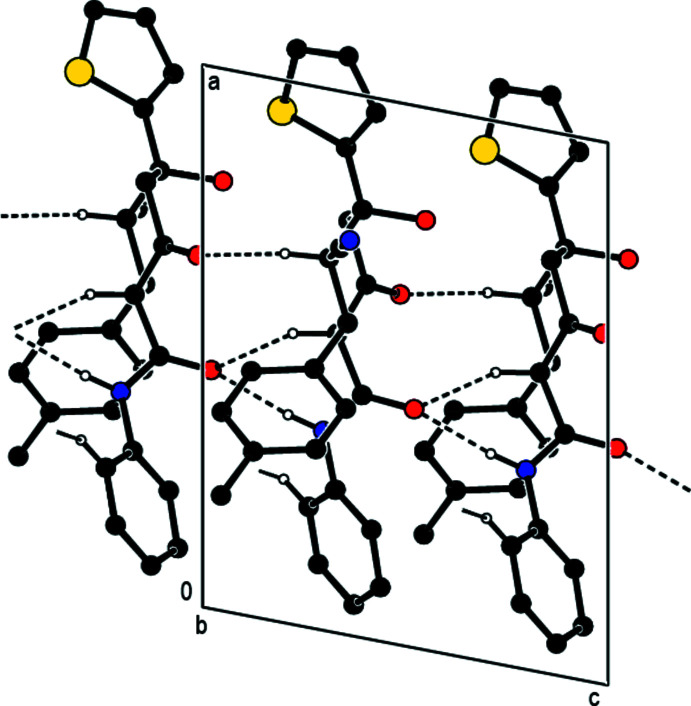
A view down the *b* axis of the inter­molecular N—H⋯O, C—H⋯O and C—H⋯N hydrogen bonds of the title compound.

**Table 1:** Hydrogen-bond geometry (Å, °)

*D*—H⋯*A*	*D*—H	H⋯*A*	*D*⋯*A*	*D*—H⋯*A*
N1—H1*N*⋯O2^i^	0.89 (2)	2.00 (2)	2.886 (2)	174 (2)
C2—H2⋯O2^i^	1.00	2.44	3.320 (2)	146
C4—H4⋯O1^i^	1.00	2.54	3.434 (2)	149
C9—H9⋯N2^ii^	0.95	2.57	3.272 (3)	131

Symmetry codes: (i) x, -y+{\script{1\over 2}}, z-{\script{1\over 2}}; (ii) -x+1, y-{\script{1\over 2}}, -z+{\script{1\over 2}}.

**Table 2:** Experimental details

Crystal data	
Chemical formula	C_25_H_22_N_2_O_3_S·0.04H_2_O
*M*_r_	1724.87
Crystal system, space group	Monoclinic, *P*2_1_/*c*
Temperature (K)	100
*a*, *b*, *c* (Å)	12.049 (2), 20.223 (4), 9.1743 (18)
β (°)	100.91 (3)
*V* (Å^3^)	2195.0 (8)
*Z*	1
Radiation type	Mo *K*α
μ (mm^−1^)	0.18
Crystal size (mm)	0.36 × 0.03 × 0.03

Data collection	
Diffractometer	Bruker D8 QUEST PHOTON-III CCD
Absorption correction	Multi-scan (*SADABS*; Krause *et al.*, 2015 ▸)
*T*_min_, *T*_max_	0.930, 0.990
No. of measured, independent and observed [*I* > 2σ(*I*)] reflections	40890, 4492, 3208
*R*_int_	0.086
(sin θ/λ)_max_ (Å^−1^)	0.625

Refinement	
*R*[*F* ^2^ > 2σ(*F* ^2^)], *wR*(*F* ^2^), *S*	0.043, 0.099, 1.02
No. of reflections	4492
No. of parameters	297
No. of restraints	7
H-atom treatment	H atoms treated by a mixture of independent and constrained refinement
Δρ_max_, Δρ_min_ (e Å^−3^)	0.24, −0.28

Computer programs: *APEX3* (Bruker, 2018 ▸), *SAINT* (Bruker, 2013 ▸), *SHELXT2014/5* (Sheldrick, 2015*a* ▸), *SHELXL2018/3* (Sheldrick, 2015*b* ▸), *ORTEP-3 for Windows* (Farrugia, 2012 ▸) and *PLATON* (Spek, 2020 ▸).

## supplementary crystallographic information

### Crystal data

**Table:**

C_25_H_22_N_2_O_3_S·0.04H_2_O	*F*(000) = 906
*M**_r_* = 1724.87	*D*_x_ = 1.305 Mg m^−^^3^
Monoclinic, *P*2_1_/*c*	Mo *K*α radiation, λ = 0.71073 Å
*a* = 12.049 (2) Å	Cell parameters from 6355 reflections
*b* = 20.223 (4) Å	θ = 2.5–27.2°
*c* = 9.1743 (18) Å	µ = 0.18 mm^−^^1^
β = 100.91 (3)°	*T* = 100 K
*V* = 2195.0 (8) Å^3^	Needle, colourless
*Z* = 1	0.36 × 0.03 × 0.03 mm

### Data collection

**Table:**

Bruker D8 QUEST PHOTON-III CCD diffractometer	3208 reflections with *I* > 2σ(*I*)
φ and ω scans	*R*_int_ = 0.086
Absorption correction: multi-scan (SADABS; Krause *et al.*, 2015)	θ_max_ = 26.4°, θ_min_ = 2.0°
*T*_min_ = 0.930, *T*_max_ = 0.990	*h* = −15→15
40890 measured reflections	*k* = −25→25
4492 independent reflections	*l* = −11→11

### Refinement

**Table:**

Refinement on *F*^2^	7 restraints
Least-squares matrix: full	Hydrogen site location: mixed
*R*[*F*^2^ > 2σ(*F*^2^)] = 0.043	H atoms treated by a mixture of independent and constrained refinement
*wR*(*F*^2^) = 0.099	*w* = 1/[σ^2^(*F*_o_^2^) + (0.034*P*)^2^ + 1.3559*P*] where *P* = (*F*_o_^2^ + 2*F*_c_^2^)/3
*S* = 1.02	(Δ/σ)_max_ < 0.001
4492 reflections	Δρ_max_ = 0.24 e Å^−^^3^
297 parameters	Δρ_min_ = −0.28 e Å^−^^3^

### Special details

**Table:**

Geometry. All esds (except the esd in the dihedral angle between two l.s. planes) are estimated using the full covariance matrix. The cell esds are taken into account individually in the estimation of esds in distances, angles and torsion angles; correlations between esds in cell parameters are only used when they are defined by crystal symmetry. An approximate (isotropic) treatment of cell esds is used for estimating esds involving l.s. planes.

### Fractional atomic coordinates and isotropic or equivalent isotropic displacement parameters (Å^2^)

**Table:**

	*x*	*y*	*z*	*U*_iso_*/*U*_eq_	Occ. (<1)
S1	0.94322 (4)	0.33798 (3)	0.19635 (6)	0.02478 (14)	
O1	0.64646 (13)	0.20448 (7)	0.48755 (16)	0.0284 (4)	
O2	0.44002 (12)	0.28766 (7)	0.52178 (15)	0.0236 (3)	
O3	0.79249 (12)	0.35394 (8)	0.55025 (15)	0.0262 (4)	
H3O	0.8523 (16)	0.3394 (12)	0.595 (3)	0.039*	
O4	0.612 (3)	0.351 (2)	0.700 (4)	0.031 (14)	0.040 (5)
N1	0.36773 (14)	0.23974 (9)	0.29809 (18)	0.0206 (4)	
H1N	0.3849 (18)	0.2305 (11)	0.211 (3)	0.025*	
N2	0.72836 (16)	0.51020 (9)	0.3630 (2)	0.0281 (4)	
C1	0.65290 (17)	0.24998 (10)	0.4037 (2)	0.0209 (4)	
C2	0.55201 (16)	0.29125 (10)	0.3322 (2)	0.0184 (4)	
H2	0.536629	0.282554	0.222891	0.022*	
C3	0.57511 (16)	0.36608 (10)	0.3578 (2)	0.0185 (4)	
H3	0.582369	0.375121	0.466358	0.022*	
C4	0.68857 (16)	0.38432 (9)	0.3124 (2)	0.0180 (4)	
H4	0.680962	0.376165	0.203480	0.022*	
C5	0.78937 (17)	0.34269 (10)	0.3945 (2)	0.0207 (4)	
C6	0.76359 (17)	0.26933 (10)	0.3603 (2)	0.0221 (5)	
H6A	0.759197	0.261182	0.252955	0.026*	
H6B	0.825432	0.241803	0.415728	0.026*	
C7	0.44750 (17)	0.27263 (10)	0.3935 (2)	0.0190 (4)	
C8	0.26345 (17)	0.21588 (10)	0.3278 (2)	0.0198 (4)	
C9	0.22262 (17)	0.15683 (10)	0.2617 (2)	0.0230 (4)	
H9	0.264590	0.133517	0.200325	0.028*	
C10	0.12021 (18)	0.13192 (11)	0.2855 (2)	0.0272 (5)	
H10	0.092562	0.091213	0.241189	0.033*	
C11	0.05831 (18)	0.16603 (12)	0.3732 (2)	0.0284 (5)	
H11	−0.012143	0.149162	0.388622	0.034*	
C12	0.09986 (18)	0.22518 (11)	0.4386 (2)	0.0280 (5)	
H12	0.057482	0.248606	0.499304	0.034*	
C13	0.20247 (17)	0.25057 (11)	0.4167 (2)	0.0235 (5)	
H13	0.230470	0.291052	0.461766	0.028*	
C14	0.47911 (16)	0.40798 (10)	0.2754 (2)	0.0194 (4)	
C15	0.45216 (18)	0.40895 (11)	0.1203 (2)	0.0246 (5)	
H15	0.492962	0.381703	0.064508	0.030*	
C16	0.36664 (18)	0.44924 (11)	0.0475 (2)	0.0269 (5)	
H16	0.349713	0.449300	−0.057939	0.032*	
C17	0.30501 (17)	0.48957 (11)	0.1251 (2)	0.0256 (5)	
C18	0.33140 (18)	0.48823 (11)	0.2794 (2)	0.0257 (5)	
H18	0.290300	0.515437	0.334897	0.031*	
C19	0.41680 (17)	0.44777 (10)	0.3536 (2)	0.0219 (4)	
H19	0.432838	0.447282	0.459033	0.026*	
C20	0.2125 (2)	0.53442 (12)	0.0457 (3)	0.0349 (6)	
H20A	0.188806	0.519550	−0.057203	0.052*	
H20B	0.240883	0.579854	0.046566	0.052*	
H20C	0.147738	0.532911	0.096157	0.052*	
C21	0.71119 (17)	0.45522 (11)	0.3399 (2)	0.0216 (4)	
C22	0.89866 (17)	0.36488 (10)	0.3544 (2)	0.0218 (4)	
C23	0.97306 (17)	0.40966 (11)	0.4282 (2)	0.0267 (5)	
H23	0.962924	0.430925	0.517123	0.032*	
C24	1.06711 (19)	0.42148 (12)	0.3596 (3)	0.0312 (5)	
H24	1.127012	0.450845	0.398265	0.037*	
C25	1.06228 (18)	0.38632 (11)	0.2328 (2)	0.0280 (5)	
H25	1.117772	0.388191	0.171907	0.034*	

### Atomic displacement parameters (Å^2^)

**Table:**

	*U*^11^	*U*^22^	*U*^33^	*U*^12^	*U*^13^	*U*^23^
S1	0.0239 (3)	0.0333 (3)	0.0184 (3)	−0.0043 (2)	0.0072 (2)	−0.0021 (2)
O1	0.0322 (8)	0.0279 (8)	0.0260 (8)	0.0009 (7)	0.0077 (7)	0.0086 (7)
O2	0.0275 (8)	0.0310 (8)	0.0141 (7)	−0.0036 (6)	0.0085 (6)	−0.0010 (6)
O3	0.0262 (8)	0.0395 (10)	0.0130 (7)	0.0029 (7)	0.0038 (6)	0.0014 (6)
O4	0.024 (18)	0.04 (2)	0.027 (19)	−0.001 (13)	−0.004 (13)	−0.009 (13)
N1	0.0234 (9)	0.0270 (10)	0.0130 (8)	−0.0028 (8)	0.0077 (7)	−0.0021 (7)
N2	0.0329 (10)	0.0281 (11)	0.0235 (10)	−0.0047 (8)	0.0060 (8)	−0.0014 (8)
C1	0.0272 (11)	0.0221 (11)	0.0143 (10)	−0.0014 (9)	0.0062 (8)	−0.0013 (8)
C2	0.0211 (10)	0.0236 (11)	0.0112 (9)	−0.0022 (8)	0.0050 (8)	−0.0014 (8)
C3	0.0218 (10)	0.0210 (10)	0.0138 (9)	−0.0012 (8)	0.0064 (8)	−0.0015 (8)
C4	0.0204 (10)	0.0207 (10)	0.0132 (9)	−0.0023 (8)	0.0038 (8)	−0.0020 (8)
C5	0.0221 (10)	0.0272 (11)	0.0131 (9)	0.0005 (9)	0.0042 (8)	0.0009 (8)
C6	0.0214 (10)	0.0247 (11)	0.0207 (11)	0.0021 (9)	0.0054 (9)	0.0036 (9)
C7	0.0219 (10)	0.0192 (10)	0.0168 (10)	0.0018 (8)	0.0063 (8)	0.0026 (8)
C8	0.0207 (10)	0.0243 (11)	0.0153 (10)	−0.0003 (8)	0.0055 (8)	0.0040 (8)
C9	0.0277 (11)	0.0245 (11)	0.0176 (10)	0.0006 (9)	0.0067 (9)	0.0001 (9)
C10	0.0300 (12)	0.0283 (12)	0.0223 (11)	−0.0057 (10)	0.0029 (9)	0.0025 (9)
C11	0.0231 (11)	0.0386 (13)	0.0245 (11)	−0.0061 (10)	0.0065 (9)	0.0049 (10)
C12	0.0271 (11)	0.0361 (13)	0.0228 (11)	0.0029 (10)	0.0102 (9)	0.0013 (10)
C13	0.0248 (11)	0.0257 (11)	0.0200 (10)	0.0004 (9)	0.0046 (9)	0.0001 (9)
C14	0.0226 (10)	0.0193 (10)	0.0171 (10)	−0.0027 (8)	0.0058 (8)	0.0009 (8)
C15	0.0292 (11)	0.0275 (12)	0.0183 (10)	0.0021 (9)	0.0075 (9)	−0.0029 (9)
C16	0.0300 (12)	0.0318 (12)	0.0183 (10)	0.0044 (10)	0.0033 (9)	0.0014 (9)
C17	0.0232 (11)	0.0261 (11)	0.0277 (12)	0.0004 (9)	0.0056 (9)	0.0028 (9)
C18	0.0267 (11)	0.0255 (12)	0.0274 (11)	0.0024 (9)	0.0118 (9)	−0.0009 (9)
C19	0.0239 (10)	0.0271 (11)	0.0165 (10)	−0.0013 (9)	0.0083 (9)	−0.0028 (9)
C20	0.0335 (13)	0.0390 (14)	0.0327 (13)	0.0106 (11)	0.0075 (11)	0.0064 (11)
C21	0.0215 (10)	0.0295 (12)	0.0146 (10)	−0.0020 (9)	0.0058 (8)	0.0011 (9)
C22	0.0244 (11)	0.0250 (11)	0.0161 (10)	0.0020 (9)	0.0038 (8)	0.0016 (8)
C23	0.0256 (11)	0.0329 (13)	0.0221 (11)	−0.0007 (10)	0.0057 (9)	−0.0048 (9)
C24	0.0266 (11)	0.0360 (13)	0.0310 (12)	−0.0065 (10)	0.0056 (10)	−0.0020 (10)
C25	0.0221 (11)	0.0377 (13)	0.0253 (11)	−0.0059 (10)	0.0076 (9)	0.0011 (10)

### Geometric parameters (Å, º)

**Table:**

S1—C25	1.716 (2)	C9—H9	0.9500
S1—C22	1.727 (2)	C10—C11	1.381 (3)
O1—C1	1.211 (2)	C10—H10	0.9500
O2—C7	1.236 (2)	C11—C12	1.389 (3)
O3—C5	1.440 (2)	C11—H11	0.9500
O3—H3O	0.815 (16)	C12—C13	1.388 (3)
N1—C7	1.347 (3)	C12—H12	0.9500
N1—C8	1.420 (3)	C13—H13	0.9500
N1—H1N	0.89 (2)	C14—C19	1.389 (3)
N2—C21	1.143 (3)	C14—C15	1.398 (3)
C1—C6	1.514 (3)	C15—C16	1.382 (3)
C1—C2	1.517 (3)	C15—H15	0.9500
C2—C7	1.520 (3)	C16—C17	1.387 (3)
C2—C3	1.549 (3)	C16—H16	0.9500
C2—H2	1.0000	C17—C18	1.391 (3)
C3—C14	1.515 (3)	C17—C20	1.513 (3)
C3—C4	1.547 (3)	C18—C19	1.388 (3)
C3—H3	1.0000	C18—H18	0.9500
C4—C21	1.472 (3)	C19—H19	0.9500
C4—C5	1.551 (3)	C20—H20A	0.9800
C4—H4	1.0000	C20—H20B	0.9800
C5—C22	1.501 (3)	C20—H20C	0.9800
C5—C6	1.536 (3)	C22—C23	1.361 (3)
C6—H6A	0.9900	C23—C24	1.417 (3)
C6—H6B	0.9900	C23—H23	0.9500
C8—C13	1.387 (3)	C24—C25	1.355 (3)
C8—C9	1.387 (3)	C24—H24	0.9500
C9—C10	1.388 (3)	C25—H25	0.9500
			
C25—S1—C22	92.19 (11)	C11—C10—H10	119.8
C5—O3—H3O	107.5 (18)	C9—C10—H10	119.8
C7—N1—C8	126.29 (17)	C10—C11—C12	119.5 (2)
C7—N1—H1N	115.6 (14)	C10—C11—H11	120.3
C8—N1—H1N	118.0 (14)	C12—C11—H11	120.3
O1—C1—C6	121.94 (19)	C13—C12—C11	120.9 (2)
O1—C1—C2	123.36 (19)	C13—C12—H12	119.5
C6—C1—C2	114.70 (17)	C11—C12—H12	119.5
C1—C2—C7	110.84 (16)	C8—C13—C12	118.9 (2)
C1—C2—C3	111.44 (16)	C8—C13—H13	120.5
C7—C2—C3	108.88 (16)	C12—C13—H13	120.5
C1—C2—H2	108.5	C19—C14—C15	118.25 (19)
C7—C2—H2	108.5	C19—C14—C3	120.15 (17)
C3—C2—H2	108.5	C15—C14—C3	121.59 (18)
C14—C3—C4	111.24 (16)	C16—C15—C14	120.5 (2)
C14—C3—C2	111.86 (16)	C16—C15—H15	119.7
C4—C3—C2	109.56 (16)	C14—C15—H15	119.7
C14—C3—H3	108.0	C15—C16—C17	121.4 (2)
C4—C3—H3	108.0	C15—C16—H16	119.3
C2—C3—H3	108.0	C17—C16—H16	119.3
C21—C4—C3	109.29 (16)	C16—C17—C18	118.0 (2)
C21—C4—C5	110.05 (16)	C16—C17—C20	121.5 (2)
C3—C4—C5	112.98 (16)	C18—C17—C20	120.5 (2)
C21—C4—H4	108.1	C19—C18—C17	121.1 (2)
C3—C4—H4	108.1	C19—C18—H18	119.5
C5—C4—H4	108.1	C17—C18—H18	119.5
O3—C5—C22	109.67 (16)	C18—C19—C14	120.75 (19)
O3—C5—C6	108.76 (16)	C18—C19—H19	119.6
C22—C5—C6	113.04 (17)	C14—C19—H19	119.6
O3—C5—C4	105.49 (16)	C17—C20—H20A	109.5
C22—C5—C4	111.18 (16)	C17—C20—H20B	109.5
C6—C5—C4	108.40 (16)	H20A—C20—H20B	109.5
C1—C6—C5	110.49 (17)	C17—C20—H20C	109.5
C1—C6—H6A	109.6	H20A—C20—H20C	109.5
C5—C6—H6A	109.6	H20B—C20—H20C	109.5
C1—C6—H6B	109.6	N2—C21—C4	179.1 (2)
C5—C6—H6B	109.6	C23—C22—C5	127.05 (19)
H6A—C6—H6B	108.1	C23—C22—S1	110.32 (16)
O2—C7—N1	124.66 (19)	C5—C22—S1	122.57 (15)
O2—C7—C2	120.42 (18)	C22—C23—C24	113.4 (2)
N1—C7—C2	114.92 (17)	C22—C23—H23	123.3
C13—C8—C9	120.61 (19)	C24—C23—H23	123.3
C13—C8—N1	121.85 (19)	C25—C24—C23	112.6 (2)
C9—C8—N1	117.52 (18)	C25—C24—H24	123.7
C8—C9—C10	119.7 (2)	C23—C24—H24	123.7
C8—C9—H9	120.1	C24—C25—S1	111.40 (17)
C10—C9—H9	120.1	C24—C25—H25	124.3
C11—C10—C9	120.3 (2)	S1—C25—H25	124.3
			
O1—C1—C2—C7	6.3 (3)	C8—C9—C10—C11	0.7 (3)
C6—C1—C2—C7	−174.55 (16)	C9—C10—C11—C12	−0.6 (3)
O1—C1—C2—C3	127.8 (2)	C10—C11—C12—C13	0.2 (3)
C6—C1—C2—C3	−53.1 (2)	C9—C8—C13—C12	0.1 (3)
C1—C2—C3—C14	174.89 (16)	N1—C8—C13—C12	178.60 (19)
C7—C2—C3—C14	−62.5 (2)	C11—C12—C13—C8	0.0 (3)
C1—C2—C3—C4	51.1 (2)	C4—C3—C14—C19	−120.6 (2)
C7—C2—C3—C4	173.62 (15)	C2—C3—C14—C19	116.5 (2)
C14—C3—C4—C21	57.0 (2)	C4—C3—C14—C15	58.1 (2)
C2—C3—C4—C21	−178.84 (15)	C2—C3—C14—C15	−64.8 (2)
C14—C3—C4—C5	179.86 (16)	C19—C14—C15—C16	0.8 (3)
C2—C3—C4—C5	−55.9 (2)	C3—C14—C15—C16	−177.84 (19)
C21—C4—C5—O3	64.9 (2)	C14—C15—C16—C17	−0.2 (3)
C3—C4—C5—O3	−57.6 (2)	C15—C16—C17—C18	−0.3 (3)
C21—C4—C5—C22	−54.0 (2)	C15—C16—C17—C20	179.1 (2)
C3—C4—C5—C22	−176.43 (16)	C16—C17—C18—C19	0.0 (3)
C21—C4—C5—C6	−178.80 (16)	C20—C17—C18—C19	−179.4 (2)
C3—C4—C5—C6	58.7 (2)	C17—C18—C19—C14	0.7 (3)
O1—C1—C6—C5	−124.8 (2)	C15—C14—C19—C18	−1.1 (3)
C2—C1—C6—C5	56.1 (2)	C3—C14—C19—C18	177.61 (18)
O3—C5—C6—C1	57.7 (2)	O3—C5—C22—C23	−22.9 (3)
C22—C5—C6—C1	179.77 (16)	C6—C5—C22—C23	−144.4 (2)
C4—C5—C6—C1	−56.5 (2)	C4—C5—C22—C23	93.4 (2)
C8—N1—C7—O2	−1.1 (3)	O3—C5—C22—S1	160.44 (14)
C8—N1—C7—C2	178.99 (18)	C6—C5—C22—S1	38.9 (2)
C1—C2—C7—O2	71.2 (2)	C4—C5—C22—S1	−83.3 (2)
C3—C2—C7—O2	−51.7 (2)	C25—S1—C22—C23	0.82 (17)
C1—C2—C7—N1	−108.87 (19)	C25—S1—C22—C5	178.01 (18)
C3—C2—C7—N1	128.20 (18)	C5—C22—C23—C24	−178.2 (2)
C7—N1—C8—C13	36.3 (3)	S1—C22—C23—C24	−1.2 (2)
C7—N1—C8—C9	−145.1 (2)	C22—C23—C24—C25	1.0 (3)
C13—C8—C9—C10	−0.5 (3)	C23—C24—C25—S1	−0.4 (3)
N1—C8—C9—C10	−179.04 (18)	C22—S1—C25—C24	−0.26 (19)

### Hydrogen-bond geometry (Å, º)

**Table:**

*D*—H···*A*	*D*—H	H···*A*	*D*···*A*	*D*—H···*A*
N1—H1*N*···O2^i^	0.89 (2)	2.00 (2)	2.886 (2)	174 (2)
C2—H2···O2^i^	1.00	2.44	3.320 (2)	146
C3—H3···O3	1.00	2.54	2.879 (2)	100
C4—H4···O1^i^	1.00	2.54	3.434 (2)	149
C6—H6*A*···S1	0.99	2.83	3.179 (2)	101
C9—H9···N2^ii^	0.95	2.57	3.272 (3)	131
C13—H13···O2	0.95	2.48	2.939 (3)	110

Symmetry codes: (i) *x*, −*y*+1/2, *z*−1/2; (ii) −*x*+1, *y*−1/2, −*z*+1/2.
