# Supplementary figures and images for: Crystal structure and Hirshfeld surface analysis of 3-cyano-4-hy­droxy-2-(4-methyl­phen­yl)-6-oxo-N-phenyl-4-(thio­phen-2-yl)cyclo­hexane-1-carbox­amide 0.04-hydrate
Source: Acta Crystallogr E Crystallogr Commun. 2021 Mar 9;77(Pt 4):366–71. doi: 10.1107/S2056989021002449 (PMC8025867; doi:10.1107/S2056989021002449)

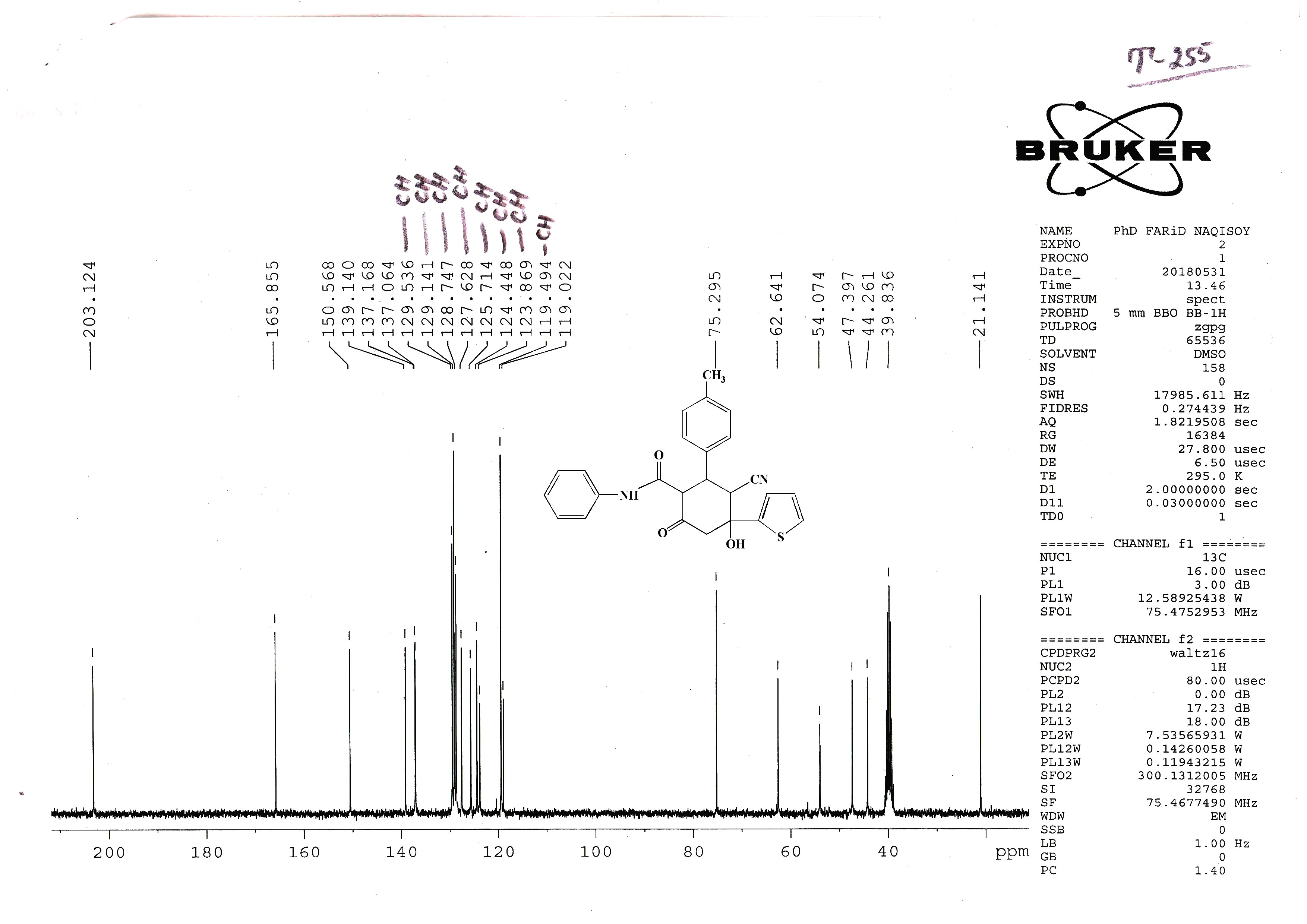

Supplement: Supplementary file 3 [file e-77-00366-sup3.jpg]

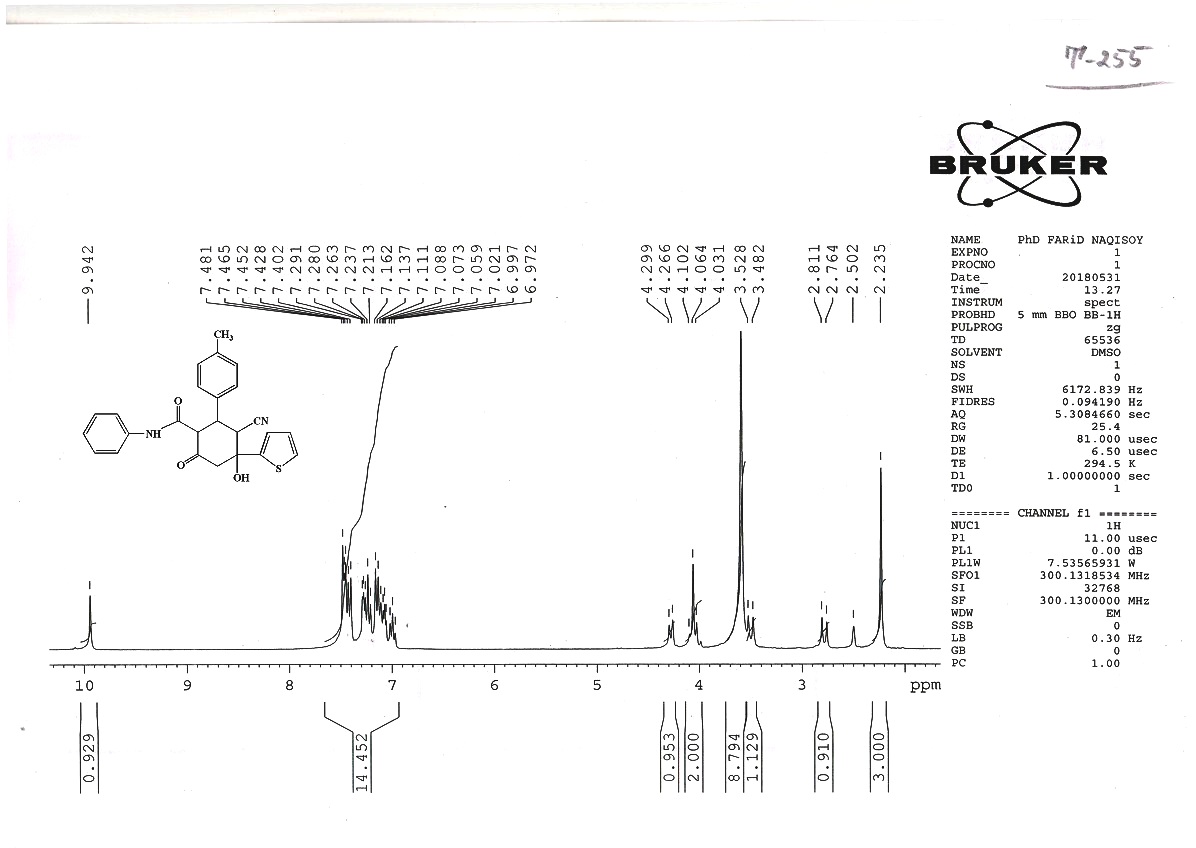

Supplement: Supplementary file 4 [file e-77-00366-sup4.jpg]
